# A modified multiplex ligation-dependent probe amplification method for the detection of 22q11.2 copy number variations in patients with congenital heart disease

**DOI:** 10.1186/s12864-015-1590-5

**Published:** 2015-05-08

**Authors:** Xiaoqing Zhang, Yuejuan Xu, Deyuan Liu, Juan Geng, Sun Chen, Zhengwen Jiang, Qihua Fu, Kun Sun

**Affiliations:** Department of Laboratory Medicine, Shanghai Children’s Medical Center, Shanghai Jiao Tong University School of Medicine, Shanghai, 200127 Peoples Republic of China; Department of Pediatric Cardiology, Xinhua Hospital, Shanghai Jiao Tong University School of Medicine, Shanghai, 200092 Peoples Republic of China; Genesky Diagnostics (Suzhou) Inc, Suzhou, Peoples Republic of China

**Keywords:** Copy number variation, 22q11.2 deletion, Congenital heart disease, Multiplex ligation-dependent probe amplification

## Abstract

**Background:**

Copy number variations (CNVs) of chromosomal region 22q11.2 are associated with a subset of patients with congenital heart disease (CHD). Accurate and efficient detection of CNV is important for genetic analysis of CHD. The aim of the study was to introduce a novel approach named CNVplex®, a high-throughput analysis technique designed for efficient detection of chromosomal CNVs, and to explore the prevalence of sub-chromosomal imbalances in 22q11.2 loci in patients with CHD from a single institute.

**Results:**

We developed a novel technique, CNVplex®, for high-throughput detection of sub-chromosomal copy number aberrations. Modified from the multiplex ligation-dependent probe amplification (MLPA) method, it introduced a lengthening ligation system and four universal primer sets, which simplified the synthesis of probes and significantly improved the flexibility of the experiment. We used 110 samples, which were extensively characterized with chromosomal microarray analysis and MLPA, to validate the performance of the newly developed method. Furthermore, CNVplex® was used to screen for sub-chromosomal imbalances in 22q11.2 loci in 818 CHD patients consecutively enrolled from Shanghai Children’s Medical Center. In the methodology development phase, CNVplex® detected all copy number aberrations that were previously identified with both chromosomal microarray analysis and MLPA, demonstrating 100% sensitivity and specificity. In the validation phase, 22q11.2 deletion and 22q11.2 duplication were detected in 39 and 1 of 818 patients with CHD by CNVplex®, respectively. Our data demonstrated that the frequency of 22q11.2 deletion varied among sub-groups of CHD patients. Notably, 22q11.2 deletion was more commonly observed in cases with conotruncal defect (CTD) than in cases with non-CTD (P < 0.001). With higher resolution and more probes against selected chromosomal loci, CNVplex® also identified several individuals with small CNVs and alterations in other chromosomes.

**Conclusions:**

CNVplex® is sensitive and specific in its detection of CNVs, and it is an alternative to MLPA for batch screening of pathogenetic CNVs in known genomic loci.

**Electronic supplementary material:**

The online version of this article (doi:10.1186/s12864-015-1590-5) contains supplementary material, which is available to authorized users.

## Background

Copy number variation (CNV) of DNA sequences is a source of genetic variation and has an important role in the genetics of complex disease. Analysis of CNV in diseases has led to the identification of novel disease-causing mutations and increasingly offers important new insights into the genetics of these disorders [1,2].

The chromosome region 22q11.2 is a hotspot for genomic rearrangement and related disorders. Aberrations of this region are among the most common constitutional chromosomal abnormalities [3]. Patients with 22q11.2 deletion demonstrate a broad spectrum of clinical phenotypes. Congenital heart disease (CHD) is the most frequently observed clinical manifestation, occurring in three-quarters of patients with 22q11.2 deletion [4]. Long-term studies carried out in several research centers indicate that children with CHD are most likely to undergo examinations for CNVs on chromosome 22q11.2 [5]. The results of such genetic testing are important for clinical decision makers because patients with 22q11.2 deletion need early medical intervention. Meanwhile, a more detailed study on the mechanism of interaction between multiple genes is important to understand the exact cause of the 22q11 deletion syndrome [6].

When studying CNV-associated human diseases, accurate and efficient CNV genotyping is one of the key steps. Many techniques have been widely applied in CNV studies, such as fluorescence *in situ* hybridization, comparative genomic hybridization arrays (CGH), quantitative real-time PCR, and multiplex ligation-dependent probe amplification (MLPA). These assays rely on either array- or PCR-based methods and offer different degrees of resolution, precision, and throughput [7]. All methods have advantages and limitations in different settings.

In this paper, we present a new approach named CNVplex® for the detection of chromosomal alterations using CNVs in the 22q11.2 region as a test model. This technique can analyze up to 160 gene loci in a single array. In our report, 157 loci along the 22q11.2 region and other chromosomes whose defects led to a 22q11.2 deletion syndrome-like clinical entity were analyzed simultaneously. The performance of CNVplex® was evaluated using samples with and without known CNVs on chromosome 22 that were previously characterized by chromosomal microarray analysis (CMA) and MLPA. The assay was then applied in a clinical setting in subjects with CHD, which is a population that is highly enriched for 22q11.2 deletion.

## Methods

### Cohorts

First, 110 cases, which were previously extensively characterized by CMA and MLPA, were used to evaluate the performance of CNVplex®. Next, 818 patients with at least one congenital heart defect were enrolled from November 2011 to January 2014 in Shanghai Children’s Medical Center (Table 1). A cardiologist confirmed the CHD diagnosis for all patients by reviewing and evaluating patient history, physical examinations, and medical records. Patients with mild CHD abnormalities, e.g., isolated patent ductus arteriosus, atrial/ventricular septal defect, or patent foramen ovale, were excluded. The patients were divided into two groups: conotruncal heart defect (CTD) (n = 615) and non-CTD (n = 203). All subjects were unrelated, and the median age at the time of diagnosis was 10 months with a range of 3 days to 18 years. Peripheral blood samples of all cases were obtained, and genomic DNA was extracted using the QIAmp DNA Blood Mini Kit (Qiagen, Hilden, Germany) with standard protocols. The DNA quality was tested by optical density (OD) 260/280 nm ratios, quantified by ultraviolet spectrophotometry, and stored at −20°C until use. Written consent was obtained from parents or guardians of all patients. This study was approved by the Ethics Committee of Shanghai Children’s Medical Center.

**Table 1:** **Prevalence of 22q11.2 deletions in patients with CHD**

**Cardiac phenotypes**	**Total subjects**	**22q11.2 deletion subjects**	**Subjects frequency (%)**
**Conotruncal cardiac defects**			
Tetralogy of Fallot	231	20	8.7
Pulmonary atresia/Ventricular septal defect	135	17	12.6
Double outlet right ventricle	115		
Transposition of great arteries	91		
Tricuspid atresia	20		
Persistent truncus arteriosus	12	1	8.3
Interrupted aortic arch	11		
**Non-conotruncal cardiac defects**			
Single atrium/Single ventricle	58		
Atrioventricular septal defect	59		
Total anomalous pulmonary venous connection	30	1	3.3
Partial anomaly of pulmonary venous connection	14		
Subvalvular aortic stenosis	16		
Coarctation of the aorta	15		
Others	11		
**Total**	818	39	4.8

Atrioventricular septal defect involved complete, partial and transitional atrioventricular septal defect. Others involved Aortopulmonary window and Cor triatriatum.

### CNVplex®

**Figure 1:**
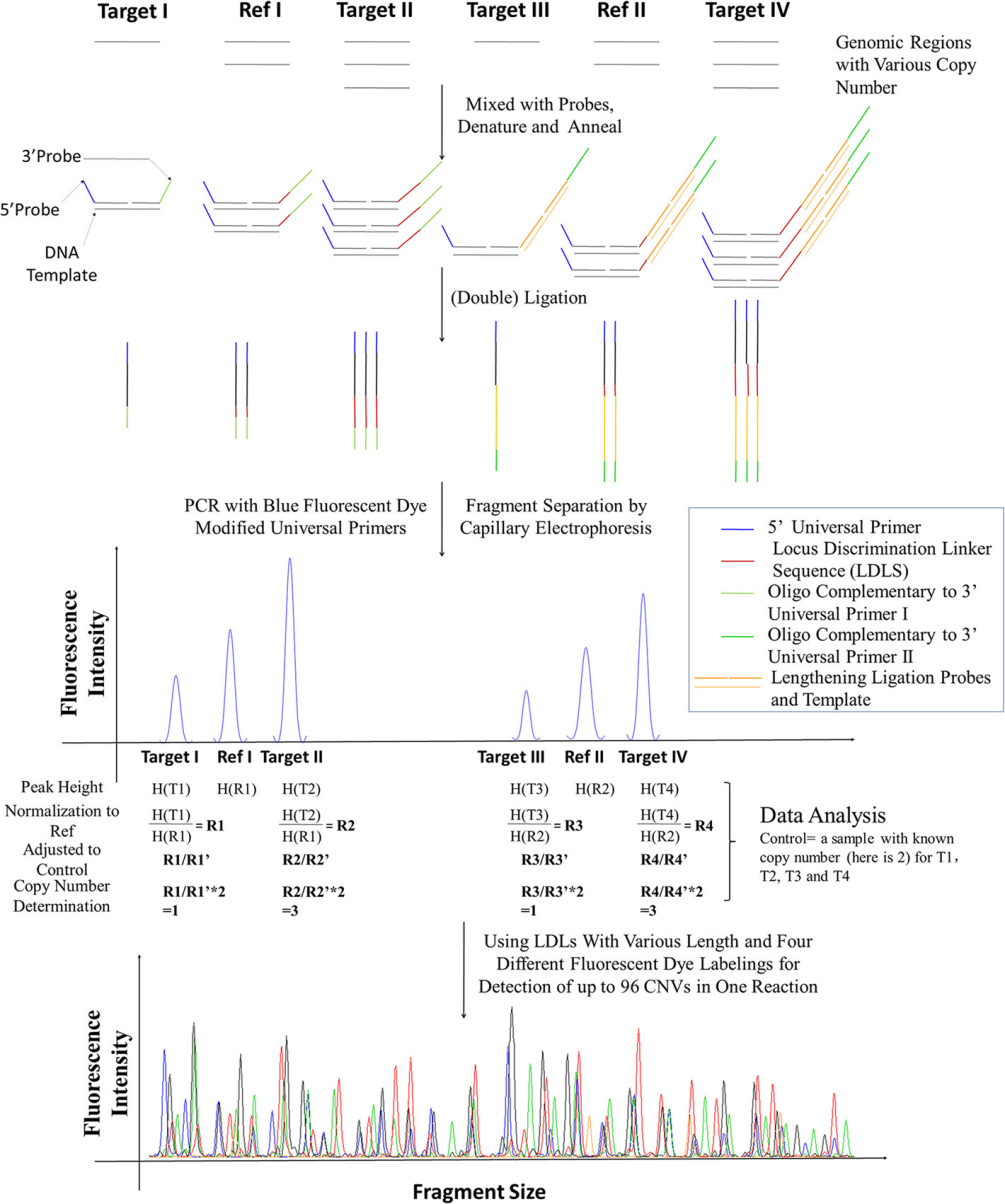
A diagram of CNVplex® technology. Two reference loci (R1/R2) and four target genomic segments (T1–T4) with various copy numbers are illustrated in this figure. They are mixed with probes, denatured, and annealed under certain conditions. The 5’ probes contain oligonucleotides specific to 5’ universal primers, and the 3’ probes include a locus discrimination linker sequence (LDLS) and oligonucleotides complementary to 3’ universal primers. Each pair of the probes contains a hybridization oligonucleotide that is specific to the human genomic DNA and can hybridize immediately adjacent to each other without a gap. The lengthening ligation system, which consists of a pair of lengthening ligation probes and a certain input template, is shown as orange lines. The probes are specific to the input template sequence and can hybridize to the template. After hybridization, probes specific to human genomic DNA and the input template are ligated at the same time using a thermally stable ligase enzyme. The PCR primers subsequently amplify the double-ligated probe, which becomes a single contiguous molecule. Amplicons with different sizes are separated by capillary electrophoresis, and the peak heights of samples are calculated and subsequently normalized to reference segments. Using LDLSs of various length and four 5’ universal primers labeled with a different fluorescent dye, CNVplex® allows 96 loci to be detected in a single PCR.

CNVplex® is based on the MLPA method, and a schematic of the experimental principle and workflow of this technique is illustrated in Figure 1 [8].

Similar to MLPA, each array of CNVplex® contains several pairs of probes. Each probe consists of a hybridization oligonucleotide that is complementary to the target and the universal PCR primer sequence to allow PCR amplification with the fluorescent primer pairs. The downstream probe of MLPA has a stuffer sequence with variable length to generate the unique length of ligated probes, and these probes are too long to be chemically synthesized [9]. CNVplex® uses the locus discrimination linker sequence with short length so chemically synthesized oligonucleotides can be used for all probes. However, the use of synthetic oligonucleotides limits the length of the probes. To bypass these limitations, CNVplex® introduces a lengthening ligation system, which contains an input template and a pair of lengthening ligation probes. Each probe contains a sequence that is complementary to the input template sequence, and these two probes can hybridize immediately adjacent to each other without a gap. After hybridization, the probes specific to human genomic DNA and the input template are ligated at the same time by a thermally stable ligase enzyme. The PCR primers subsequently amplify the double-ligated probes exponentially, which become a single contiguous molecule. This structure supersedes the stuffer sequence in MLPA, which ensures that the size of all probes is within 72 bp and that all probes are commercially available.

In addition, to increase the number of probes that can be used, we designed four types of 5’ universal primers labeled with different fluorophores and two types of 3’ universal primers that corresponded to the conventional 3’ probe and lengthening ligation probe to amplify the ligated products. The discrimination of probes was four-fold higher using the four-color probe sets and allowed 96 probes to be used in a single PCR. Amplification products were separated by size and color using capillary electrophoresis, and the relative amount of PCR product was proportional to the amount of target sequence (Figure 1). In our project, ligation products of each sample were subsequently amplified in two PCRs. In this way, we were able to combine 192 probes in a ligation reaction. Excluding the probes that served as controls, 160 loci could be analyzed simultaneously in a single assay.

Using synthetic oligonucleotides, 189 probes were selected in this study including 157 target-specific probes and 32 reference probes. For the 22q11 region, 115 probe pairs against 46 genes distributed in a genomic region of 7.5 Mb were designed. In addition, probes against other sub-chromosomal regions were synthesized and formatted into the analysis, including 4 loci on 22q13, 8 loci on 4q35, 6 loci on chromosome 8, 6 loci on chromosome 9, 10 loci on chromosome 10, and 8 loci on chromosome 17. Thirty-two reference probes were designed to target sequences located in different sub-chromosomal loci that have not been reported to have any copy number polymorphism. The sizes of the PCR fragments and target loci sequences in each reaction are listed in Additional file 1.

Reagents in our research for the denaturation, ligation, and subsequent PCR amplification were purchased from Genesky Diagnostics (Suzhou, China) and Takara (Dalian, China). First, 150 ng of genomic DNA in 8 μL of volume was denatured for 6 min at 98°C and then immediately put on ice. Hybridization and ligation were performed for about 15 h (5 cycles of 94°C for 1 min and 60°C for 3 h) by adding 4 μL probe mix and 8 μL ligation mix (5.8 μL water, 2 μL 10× ligation buffer, and 0.2 μL Taq DNA ligase). After inactivating the enzyme for 2 min at 94°C, 20 μL EDTA (20 mM) was added when the temperature reached 72°C. The subsequent PCR was prepared in 20 μL for each sample and contained 10 μL 2× PCR Master Mix, 1 μL primer mix, 7.8 μL water, and 1.2 μL ligation product. The PCR program was as follows: 95°C for 2 min; 5 cycles of 94°C for 20 s, 65°C reduced by 1°C per cycle for 40 s, and 72°C for 1.5 min; 27 cycles of 94°C for 20 s, 60°C for 40 s, and 72°C for 1.5 min; and 68°C for 60 min. The PCR products were diluted 10-fold and denatured for 5 min at 95°C before being run by capillary electrophoresis using an ABI 3130XL genetic analyzer(Applied Biosystems Inc., Foster City, CA, USA), and the raw data were analyzed by GeneMapper 4.1 (Applied Biosystems). In CNVplex®, peak ratios between 0.75 and 1.25 are considered normal. Ratios above 1.25 indicate the presence of a gain of the target sequence while ratios below 0.75 indicate a loss of the target sequence.

### MLPA

We used a commercial SALSA P250-B1 kit (MRC-Holland, Amsterdam, The Netherlands) for the MLPA assay. The P250-B1 MLPA probe mix contains 48 probes, which includes 30 that hybridize to chromosome 22q11 and the remaining 18 control probes are located on 22q13, 10p15, 8p23, 4q34-qter, 9q34.3, and 17p13.3. All procedures were carried out according to the manufacturer’s protocol, and data were visualized and analyzed with GeneMaker software (SoftGenetics, LLC, State College, PA, USA). A threshold of the peak ratio change of <0.70 was used to identify potential deletions, and a threshold of >1.30 was used to identify potential duplications.

### CMA

A genomic hybridization was performed on each sample with the CytoScan HD array (Affymetrix, Santa Clara, CA). All arrays were labeled, hybridized, and processed in the Microarray Core Facility at Shanghai Children’s Medical Center using conditions recommended by the manufacturer. After scanning, the resulting intensity files were visualized and analyzed with the Chromosome Analysis Suite software package (Affymetrix). The results were evaluated using UCSC Genome Browser March 2009 (NCBI37/hg19).

### Real-time PCR

Primers in our research were designed with Primer3 software (Table 2). All of the selected sequences were aligned against the human genome using the BLAT program to ensure specific hybridization. A total of 1 μL (100 ng/μL) genomic DNA was amplified in a reaction mixture containing 12.5 μL ExTaq (Takara), 0.4 μM each primer, 3.3 μM SYTO9 fluorescent dye (Life technologies, Carlsbad, CA, USA), and water to a total volume of 25 μL. Cycling conditions were 94°C for 30 s and then 45 cycles of 94°C for 5 s and 60°C for 34 s. Real-time quantitative PCR was performed on the Rotor-Gene Q (Qiagen) detection system. The expression level of target genes was normalized to the *HMBS* housekeeping gene. Each experiment was run in triplicate for each subject, and copy number alterations were calculated according to the 2-△△CT method.

**Table 2:** **Primers used for real-time PCR**

**Primer name**	**Primer sequence**	**Amplicon size (bp)**	**chr**	**Genomic location (GRCh37/hg19)**
HMBS-F	TGCACGGCAGCTTAACGAT	201	11	118963966-118964166
HMBS-R	AGGCAAGGCAGTCATCAAGG			
PRODH-F	GGGAAAGGAGAGTTCAGGCAG	101	22	18918663-18918763
PRODH-R	GCTTGTTGAATAGCCTCTGTCCTAG			
DGCR6-F	GTGAAGGAGTTGCCCAGGTA	132	22	18893981-18894112
DGCR6-R	TCAGCGTGGTGTAGGACAAG			
TOP3B-F	CTGGATGACTTCGAGCTGGT	162	22	22312808-22312969
TOP3B-R	TGGCACTGAAAAGAGACTGC			

### Statistical analysis

All statistical analysis was performed with the SPSS 17.0 statistical software package. The frequencies of 22q11.2 deletion in patients in the CTD and non-CTD groups were compared with Fisher’s exact test. To assess the association of small CNVs identified in our report with CHD, we compared the frequencies of these CNVs between the case and control cohorts. The control cohort was assembled from a previously published study on the distribution and characteristics of CNVs in Asian populations. A *P* value less than 0.05 in a two-sided test was considered significant throughout this paper.

## Results

### Performance of CNVplex®

The 110 patients used in this study were initially characterized by CMA and MLPA. Using these tests, 9 of the patients (Table 3) had 22q11 CNVs, and the other 101 patients were negative. We evaluated the performance of CNVplex® in the detection of selected genomic region CNVs with these extensively characterized samples. All nine of the previously identified 22q11 CNVs were detected by this method, and no false-positive results were yielded in the remaining 101 samples, indicating 100% sensitivity and specificity.

**Table 3:** **The results detected by MLPA, CMA, and CNVplex®**

	**Laboratory results**		
	**MLPA**	**CMA**	**CNVplex®**
**P1**	chr22:19171011-21351601 loss	chr22:18648866-21465659 loss	chr22:18893757-21464055 loss
**P2**	chr22:19171011-21351601 loss	chr22:18916842-21798907 loss	chr22:18893757-21464055 loss
**P3**	chr22:19171011-21351601 loss	chr22:18916842-21798907 loss	chr22:18893757-21464055 loss
**P4**	chr22:19171011-21351601 loss	chr22:18648866-21465659 gain	chr22:18893757-21464055 loss
**P5**	chr22:19171011-21351601 loss	chr22:18916842-21465662 loss	chr22:18893757-21464055 loss
**P6**	chr22:22312856-22330186 gain	chr22:22311348-22578983 gain	chr22:22312856-22330186 gain
**P7**	chr22:19171011-21351601 loss	chr22:18916842-21800797 loss	chr22:18893757-21464055 loss
	chr22:22312856-22330186 gain	chr22:22311348-22578983 gain	chr22:22312856-22330186 gain
**P8**	chr22:19171011-21351601 loss	chr22:18916842-21798907 loss	chr22:18893757-21464055 loss
**P9**	chr22:19171011-21351601 loss	chr22:18916842-21041014 loss	chr22:18893757-20940053 loss

Furthermore, we used the peak area from 10 normal samples without copy number alterations and 6 patients with 22q11.2 deletion to evaluate the signal consistency of the CNVplex® method. The coefficient of variability (CV) of the relative peak area for each oligo pair (Additional file 2) was calculated with the following formula: CV = standard deviation/average value. All probes showed a CV ratio ≤ 0.1, passing the threshold of the variability test and further demonstrating the low variance of the method.

### CNVs detected by CNVplex® in a large cohort of CHD

The utility of CNVplex® to detect selected genomic copy number aberrations in clinical molecular diagnostic settings was further tested in a large cohort of patients with CHD. Pathogenic 22q11.2 deletions were detected in 39 of 818 patients with CHD, which included 22q11.2 duplication in 1 patient, small variants of uncertain significance (VUS) in 20 patients, and sub-chromosomal copy number aberrations in genomic loci other than 22q11 in 5 patients.

**Figure 2:**
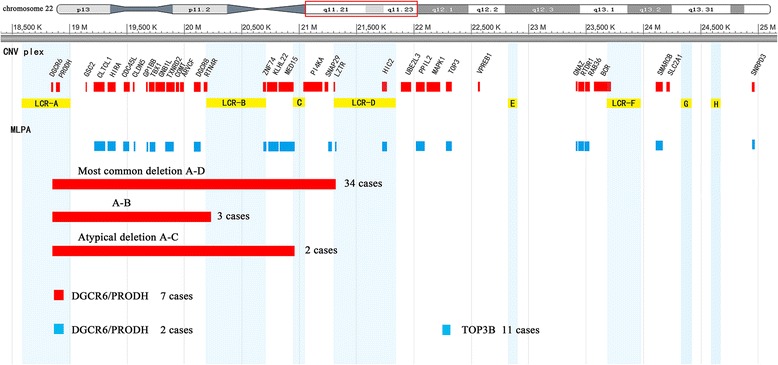
Summary of 22q11.2 copy number variations (CNVs) identified by CNVplex®. A schematic representation of the physical map of 22q11.2 showing the locations of low-copy repeats of chromosome 22 (LCR22s) (yellow boxes) and sequence position (UCSC version hg19, based on NCBI build 37). Genes that had probes in CNVplex® are shown in red boxes, and those that had probes in multiplex ligation-dependent probe amplification (MLPA) are shown in blue boxes. Solid red and blue bars below the map depict the deletions and duplication identified in this study, respectively.

Chromosome 22q11.2 is a well-characterized genomic region that contains multiple region-specific low-copy repeats (LCRs). Eight LCR clusters (LCR22A–H) are located in the proximal 22q region. For 39 patients with 22q11.2 microdeletion, the majority (34/39, 87%) shared the typical deletion flanked by LCR22A and LCR22D, spanning approximately 2.57 Mb. Three patients (3/39, 8%) had a 1.34-Mb proximally nested deletion spanning LCR22A to LCR22B, and two patients (2/39, 5%) had an atypical deletion covering LCR22A to LCR22C with a length of about 2 Mb. However, the duplication that we identified in this study using the CNVplex® technique extended distally (spanning from LCR22E to LCR22H) to the commonly deleted/duplicated region. The peak ratios of the aforementioned CNVs and typical electropherograms of the 22q11.2 deletion, 22q11.2 duplication, and normal sample are presented in Additional files 3 and 4, respectively. Using CNVplex®, we could precisely identify the breakpoints for each deletion. All deletions had a proximal breakpoint between markers *USP18* and *DGCR6*. Similarly, cases flanked distally by LCR22B had a breakpoint between *RTN4R* and *ZNF74*, whereas the deletion flanked by LCR22C had a breakpoint between *MED15* and *PI4KA* (Figure 2).

Furthermore, CNVplex® identified two VUS, including a 25-kb region containing the *DGCR6* and *PRODH* genes and a segment with *TOP3B*, respectively (Figure 2). Interestingly, 9 of 818 CHD patients harbored copy number aberrations in *DGCR6/PRODH* (7 deletions and 2 duplications), and 11 individuals had duplications in *TOP3B*.

For five patients, there were obvious changes in the probes outside the 22q11 region. There were three imbalances (two deletions and one duplication) at 4q35 and one deletion at 22q13. We also identified a complex copy number aberration at 8p23 locus in one patient, including a 10.4-Mb microdeletion immediately followed by a 2.75-Mb microduplication.

### Results of confirmatory tests

**Figure 3:**
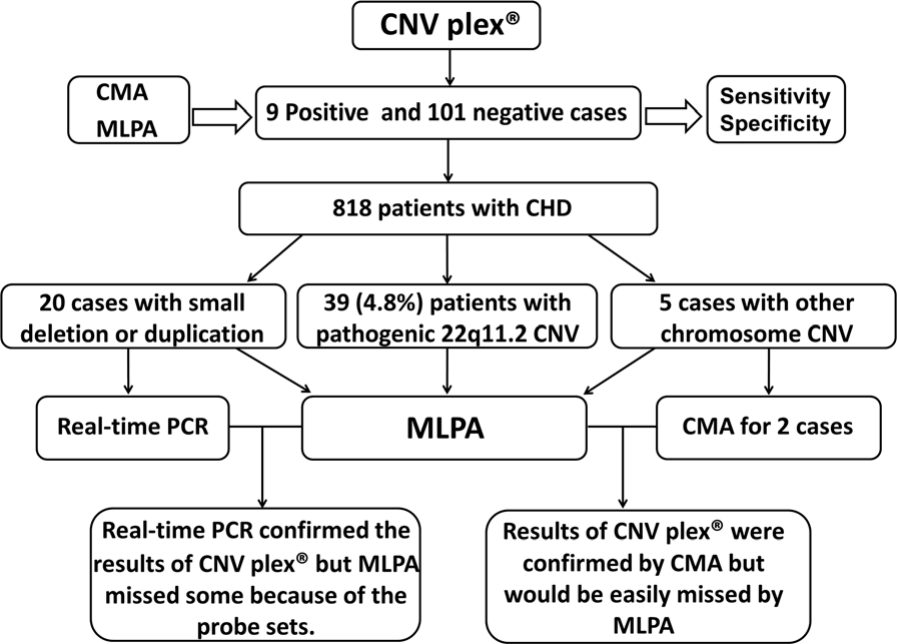
Workflow of CNV detection and validation in 818 patients with congenital heart disease.

Although there is a growing interest in the identification of CNVs, there is not a universal gold standard that is applicable for all conditions of CNV genotyping in clinical molecular diagnostic settings. The positive cases that were identified in our report were further confirmed by MLPA, CMA, or real-time PCR (Figure 3). MLPA was performed on all positive cases. CMA was used to confirm CNVs located on other chromosomes. Real-time PCR was performed for the regions that were too small to be detected by commercial MLPA kits.

**Figure 4:**
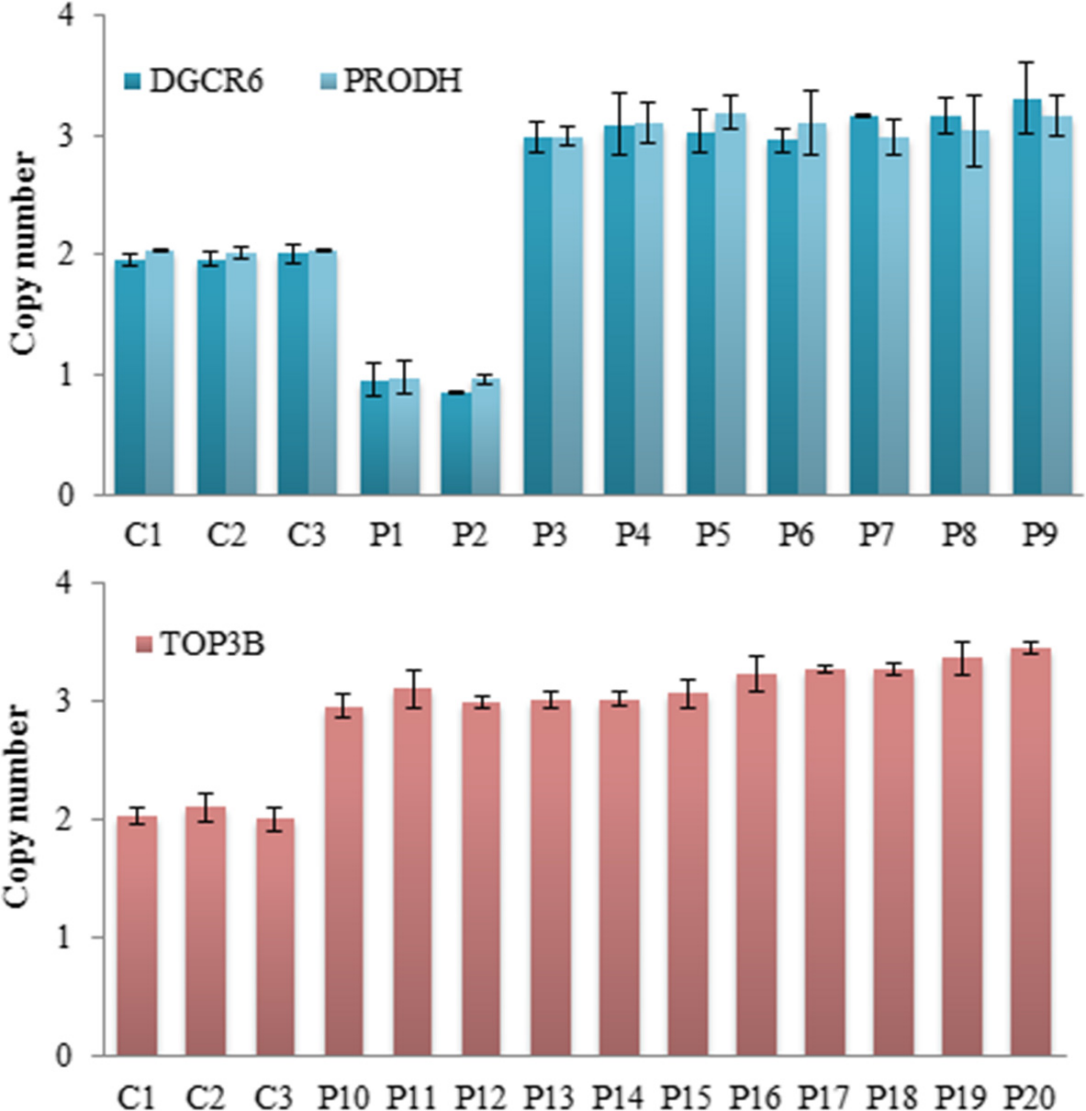
Validation of small CNVs by real-time PCR. SYTO9 and primers specific to the *DGCR6*, *PRODH*, and *TOP3B* genes were used for detection in patients (P1–P20) and control genomic DNA samples (C1–C3). The normalized gene copy number is expected to be N = 2, compared with N < 1.5 for gene deletion and N > 2.5 for duplication. The bar graph shows a 50% decrease in copy number (Y-axis) of the *DGCR6*/*PRODH* gene for patient 1 and 2 and a 33% increase (from two to three copies) for patient 3 to patient 9 (P3–P9) compared with the control samples (C1–C3). The red bars indicate a gain of one copy number (duplication) of the *TOP3B* gene in 11 patients (P10–P20) compared with the control samples (C1–C3).

**Figure 5:**
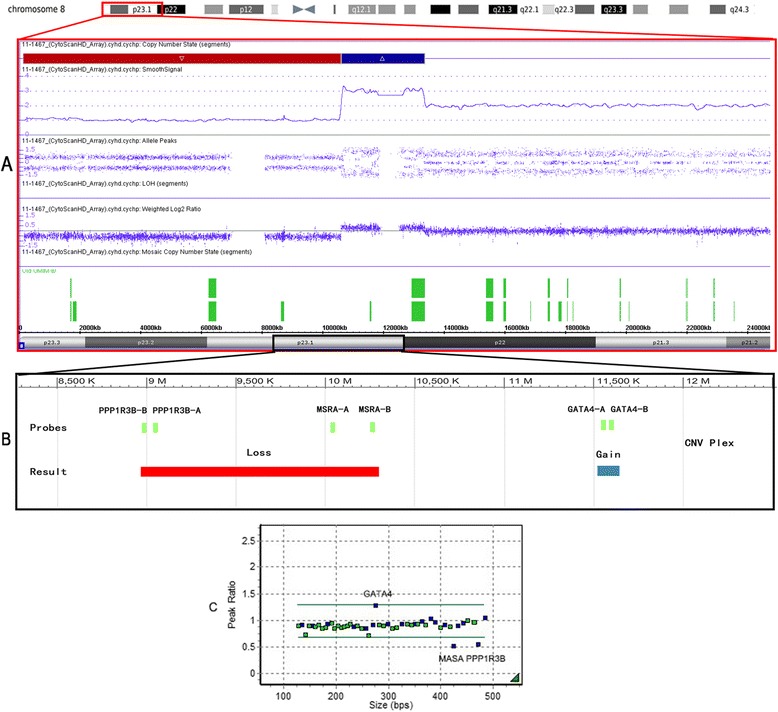
Chromosome 8 CNVs detected by chromosomal microarray, CNVplex® and MLPA. **A**. Microarray profile of chromosome 8 showing a deletion at 8pter–8p23.1 and a duplication at 8p23.1–8p22. **B**. CNVplex® probes set in 8p and results inferred from the signal. Green boxes are probes, and solid-colored bars below the map depict the results. **C**. Data of MLPA analysis. The dots represent MLPA probes, and the lines indicate the threshold.

For patients with pathogenic 22q11.2 CNVs, MLPA confirmed all of the results obtained by CNVplex® (Figure 2). However, MLPA was not optimal for cases with small CNVs because of the small number of probes against the target sequences in the MLPA B250 kit (Figure 3). For 20 patients with small CNVs, real-time PCR was performed, and the results were consistent with the copy number status ascertained by CNVplex® analyses (Figure 4). For two of the five patients with CNVs outside the 22q11 region, CMA testing was performed to precisely assess the size of the altered region. In one of the two patients analyzed with CNVs outside the 22q11 region, a deletion at 4q35 revealed by CNVplex® was confirmed by both CMA and MLPA, and a hemizygous loss of four probes located in *PPP1R3B* and *MSRA* and a gain in the signal of two probes in *GATA4* revealed by CNVplex® in the second patient was confirmed by CMA and mapped to 8p23.3–23.1 (10.4 Mb) and 8p23.1–p22 (2.75 Mb), respectively. Noticeably, MLPA also had one reference probe on each of these genes, but the probe on *GATA4* had a signal of 1.285, which was within the normal range (0.70–1.30); thus, the microduplication could be missed (Figure 5).

### Assessment of clinical manifestation

The diagnosis information and clinical data of 818 patients with CHD are summarized in Table 1. The frequency of CNVs varied between subgroups of patients with cardiac defects. Microdeletion of 22q11.2 occurred in 38 of 615 CTD patients (6.2%) and in 1 of 203 non-CTD patients (0.5%), indicating that the 22q11.2 deletion was more significantly enriched in cases with CTD than in non-CTD cases (P < 0.001).

## Discussion

Several advanced technology platforms have been frequently used in genome-wide studies on CNVs, such as CGH microarrays and next-generation sequencing. However, most of these technologies are costly, and they may not be the first choice for CNV studies of a specific gene or a few genetic loci of interest. Efficient and reliable CNV assays for target regions are important for investigating the roles of CNVs in human diseases [10]. MLPA provides a multiplex assay for copy number measurement of target DNA segments, and it has been widely used for studies on genomic disorders. However, the current MLPA design involves a long probe generated by cloning, which is a time-consuming and costly process. Additionally, MLPA is limited by the oligonucleotide length and its relatively low throughput (at most 48 loci in the same amplification reaction).

CNVplex® overcomes the intrinsic obstacles of MLPA and offers several additional advantages. The lengthening ligation system allows all probes to be manufactured by conventional oligonucleotide synthesis that circumvents the laborious step. By combining the four probe sets (labeled with different fluorophores), this approach effectively quadrupled the number of probes that can be used in an available size range. Using a group of well-characterized samples, we demonstrated that CNVplex® was an efficient, highly reliable method with high sensitivity and specificity. Further, using a cohort of CHD patients, we demonstrated the ability of the new method to correctly detect gain or loss of genomic material, to determine the candidate genes contributing to the disease, and to delineate the extent of the region involved in the rearrangement.

In the current study, CNVplex® detected common recurrent deletions (4.8% detection prevalence) in 39 individuals and a 22q11.2 duplication (0.1% duplication prevalence) in one patient among 818 CHD patients. Even though previous data suggested that the frequency of the 22q11.2 duplication was lower than that of the deletions, the estimated frequency in our report was much lower than that in other studies [11,12]. This may be due to patient selection because patients with 22q11.2 duplication have a very wide range of phenotypic variability, and the cohort with CHD was not enriched for 22q11.2 duplication.

The data enabled us to provide the prevalence of 22q11.2 deletion in defined sub-populations registered with CTD and non-CTD. Patients with CTD were more likely to have 22q11.2 deletion than non-CTD cases, 6.2% versus 0.3%, respectively (P < 0.001). Moreover, when all CHD cases were considered, pulmonary atresia (PA) was, together with ventricular septal defect (VSD), the most frequent cardiac malformation in individuals carrying the 22q11.2 deletion followed by tetralogy of Fallot (TOF) and persistent truncus arteriosus (PTA). This result was in line with the findings of previous studies, which concluded that a high prevalence of the deletion was noted in patients with TOF, PA/VSD, PTA, interrupted aortic arch, and tricuspid atresia [13,14].

It is difficult for physicians to identify 22q11.2 deletion syndrome with certainty because this syndrome shows a wide variability in phenotype expression [15]. Additionally, some characteristics such as speech difficulties and learning disabilities are not readily apparent during early infancy [16]. Because CHD is generally the first presenting symptom in such patients, molecular screening for 22q11.2 in CHD patients is important in the evaluation of associated noncardiac features [17]. The association of CTD with 22q11.2 deletion reminds us that it may be advisable to test for the microdeletion when there is a diagnosis of conotruncal malformation, especially in young infants whose extracardiac features cannot yet be excluded [18].

In addition to CNVs identified in 22q11.2, the screening of related chromosomes whose defects led to a 22q11.2 deletion syndrome-like clinical entity was crucial for CHD patients. CNVplex® uses 42 probes outside 22q11.2 according to the most-frequent chromosomal findings to optimize the diagnostic protocol [19]. The MLPA P250-B1 kit also targets those regions, but the density of probes was low. To determine more regions of interest, other MLPA kits were needed, which means more genomic DNA input and cost. In many instances, the users leave these probes out of the analysis process or simply regard them as control probes [17]. One patient in our study had an alteration in chromosome 8p. CNVplex® revealed a hemizygous loss and a subsequent gain in signal. MLPA also had reference probes on these genes, but the duplication of *GATA4* was missed. The high density of probes in CNVplex® allowed us to build more stability and reliability into the test than MLPA.

With much higher multiplexing capability, CNVplex® allowed more regions of interest to be scanned than other methods, which may provide insight into the genes or genomic regions that are crucial for specific phenotypic manifestations. Multiple dosage-sensitive genes contribute to the phenotypic spectrum of 22q11.2 deletion syndrome. One of these candidate genes is *TBX1*, whose haploinsufficiency is thought to be responsible for several phenotypes of this syndrome [20]. In this study, two variations containing the *DGCR6/PRODH* and *TOP3B* genes were identified in several individuals, and the variation in the former gene was not detected by MLPA. Xu et al. 2011 [21] created a database for common and rare CNVs in Asian populations. We compared the frequency of CNVs identified in our patient group with that of published controls. The frequency of *DGCR6/PROD*H CNVs in the patients (9 of 818 patients) was significantly higher than that in controls (2 of 6,533 individuals) (P < 0.001) and so was the duplication of *TOP3B* (11 of 818 versus 3 of 6,533 individuals, P < 0.001). Although there was no evidence for their relevance with CHD, the increased allele frequency of these genes deserved further discussion. It is likely that the identification of small CNVs within 22q11.2 will greatly contribute to our understanding of these genes and further segregation of the major regulatory genes.

In addition, using a higher density of probe sets, CNVplex® analysis could more precisely delineate the breakpoints of CNVs in selected genomic regions than MLPA. More precise determination of the breakpoints for CNVs had important implications in a study of deletion mechanisms and explanations for the high frequency of chromosome rearrangements in this region [22]. By designing probes for the unique regions interspersed within the segments containing the breakpoint region for common 22q11.2 deletion, CNVplex® allows unprecedented opportunities to define the chromosome breakpoints in individuals and to examine genotype–phenotype relationships.

## Conclusions

The lengthening ligation system and four color probe sets allow CNVplex® to detect copy number alterations of any region with higher resolution and lower cost than other methods. The successful identification of CNVs in previously well-characterized cases and CHD patients further demonstrated it to be an efficient and reliable technique. Additionally, multiple probes of CNVplex® not only allow a more exact prediction of the alterations but also make it possible to characterize typical and atypical CNVs more accurately. This offers more information than MLPA, which primarily elucidates the molecular basis of this disease and determines candidate genes. We believe that the use of CNVplex® in more individuals will yield more information.

## Additional files

Additional file 1:
**The PCR fragments sizes and target loci sequences in CNVplex®.**
Additional file 2:
**The coefficient of variability of the CNVplex® probes.**
Additional file 3:
**Peak ratios for 22q11.2 deletion/duplication patients.**
Additional file 4:
**Typical electropherograms of normal control, 22q11.2 deletion and 22q11.2 duplication.**

## Abbreviations

CNV: Copy number variation
CHD: Congenital heart disease
MLPA: Multiplex ligation-dependent probe amplification
CMA: Chromosomal microarray analysis
CGH: Comparative genomic hybridization arrays
LDLS: Locus discrimination linker sequence
CTD: Conotruncal heart defect
non-CTD: Non-conotruncal heart defect
VUS: Variants of uncertain significance
LCR: Low copy repeat
PA: Pulmonary atresia
VSD: Ventricular septal defect
TOF: Tetralogy of Fallot
PTA: Persistent truncus arteriosus
