# Supplementary material for: A modified multiplex ligation-dependent probe amplification method for the detection of 22q11.2 copy number variations in patients with congenital heart disease
Source: BMC Genomics. 2015 May 8;16(1):364. doi: 10.1186/s12864-015-1590-5 (PMC4424574; doi:10.1186/s12864-015-1590-5)
Supplement: Additional file 4: — Typical electropherograms of normal control, 22q11.2 deletion and 22q11.2 duplication. [file 12864_2015_1590_MOESM4_ESM.doc]

Additional file 4. Typical electropherograms of normal control, 22q11.2 deletion and 22q11.2 duplication. The electropherograms have a typical deletion flanked by LCR22-A and LCR22-D with a hemizygous loss of 61 loci and a duplication extended from LCR22-E to LCR22-H with a gain of one copy of 21 loci. The x axes represent fragment size in bp and y axes indicate fluorescent signals. Four kinds of fluorophores are displayed in different colors (red for PET, blue for FAM, green for VIC and yellow for NED). Genotypes for each locus were determined based on ligation-PCR product's labeling dye color and fragment size. Amplified probes are detected as fluorescent signals and peak areas are compared and normalized to determine the dosage of each target using GeneMapper 4.1 software. The loss and gain signals are indicated by red and blue arrows respectively.


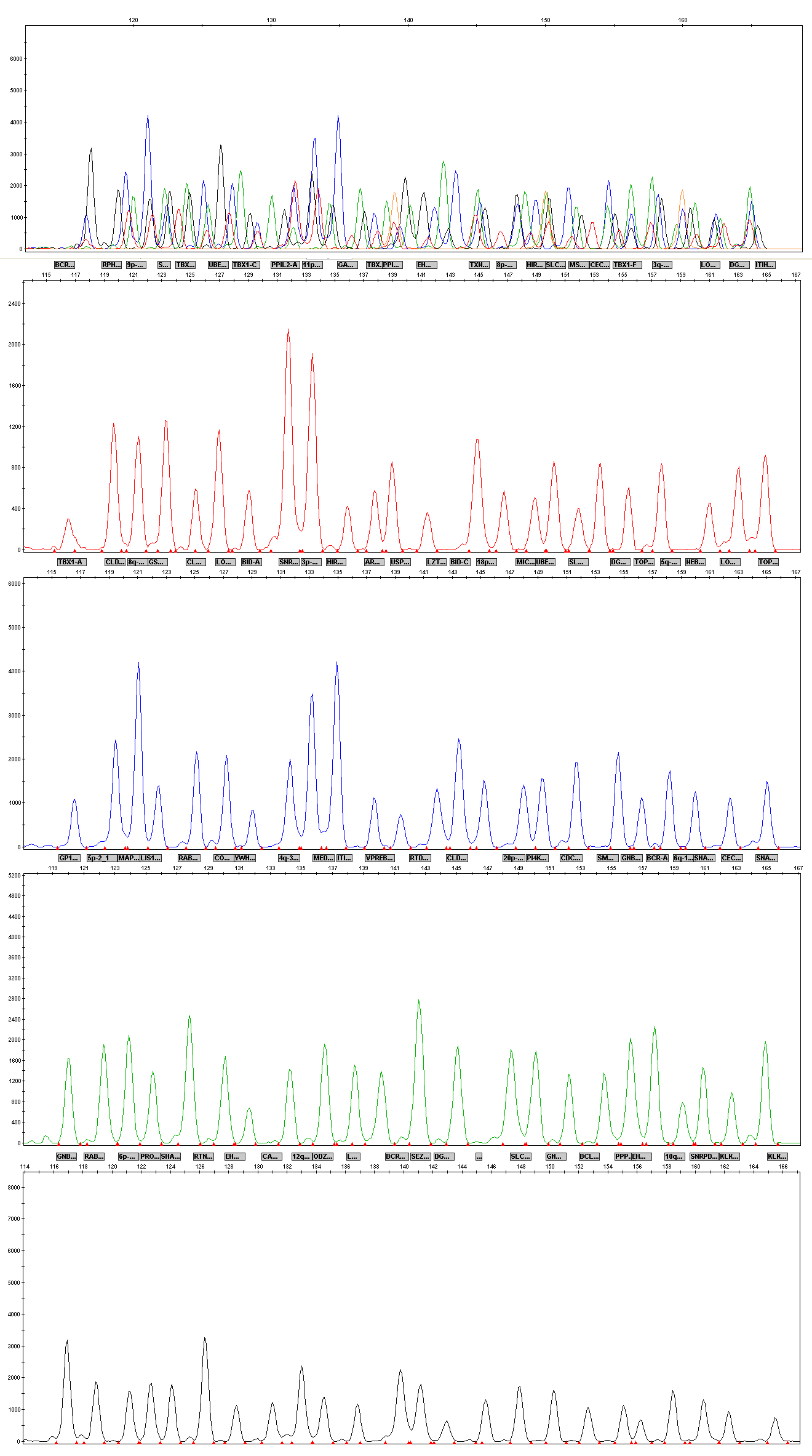


**Normal control sample-Group 1**


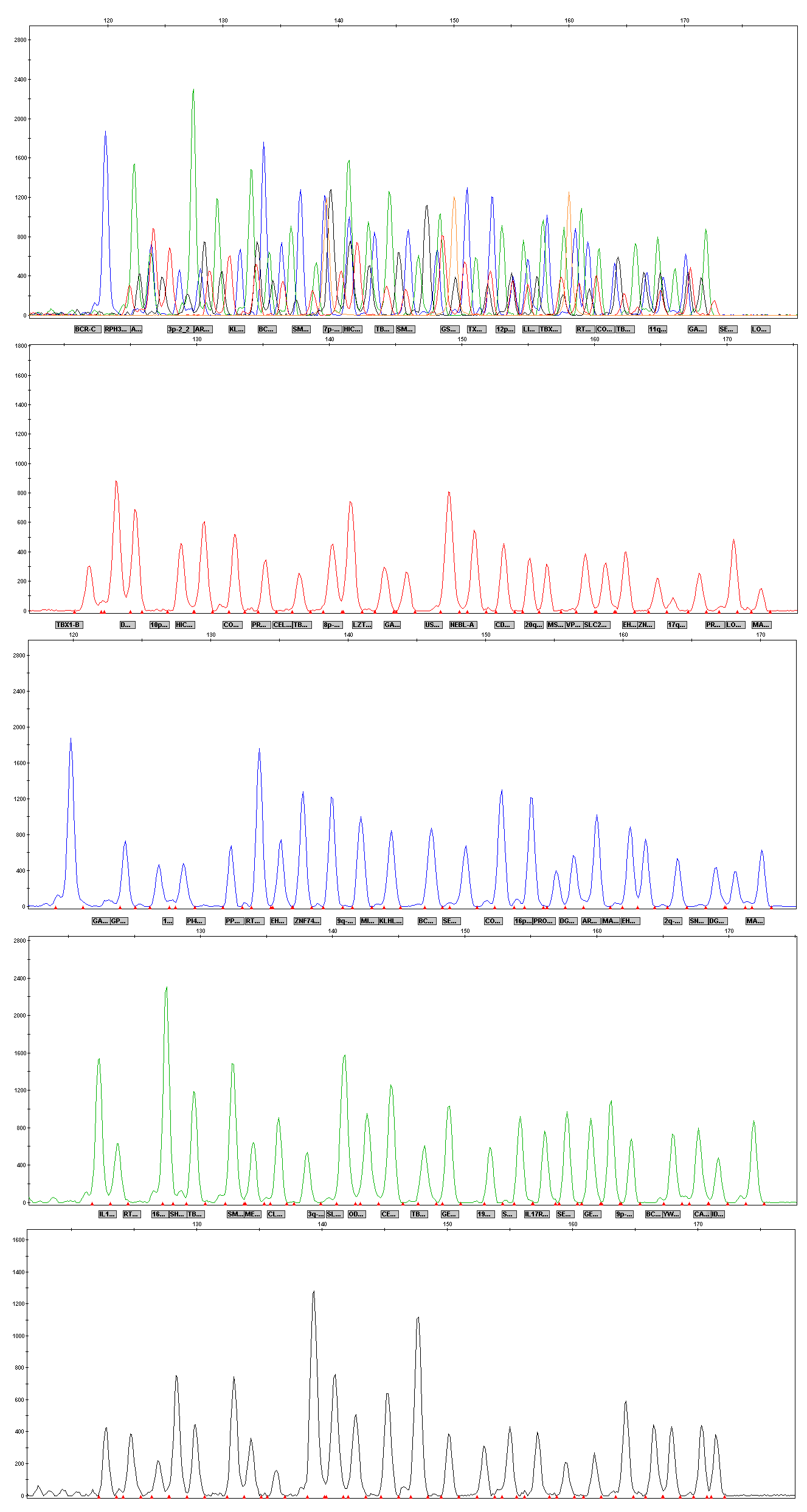


**Normal control sample-Group 2**

**
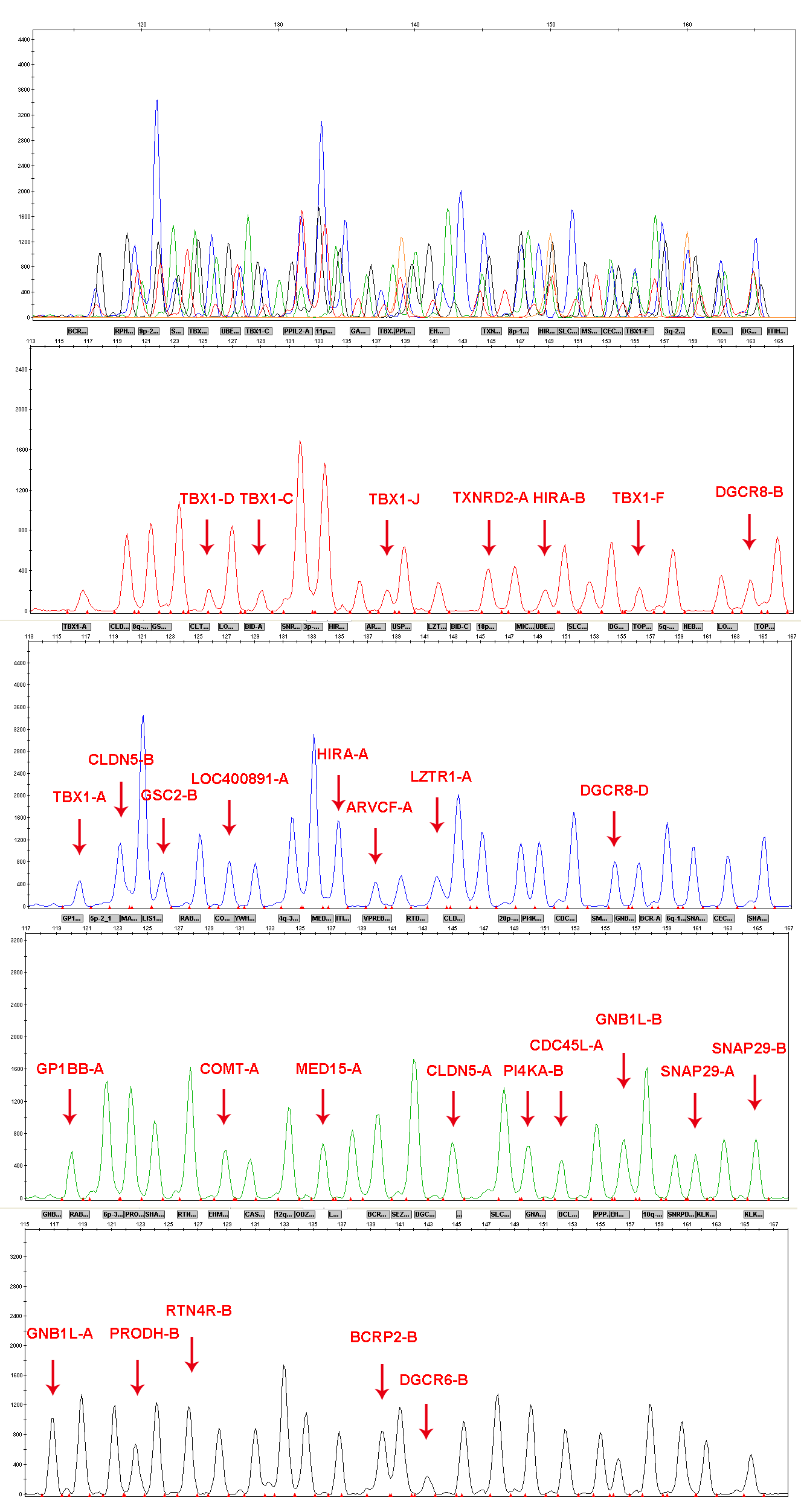
**

**22q11.2 deletion-Group 1**

**
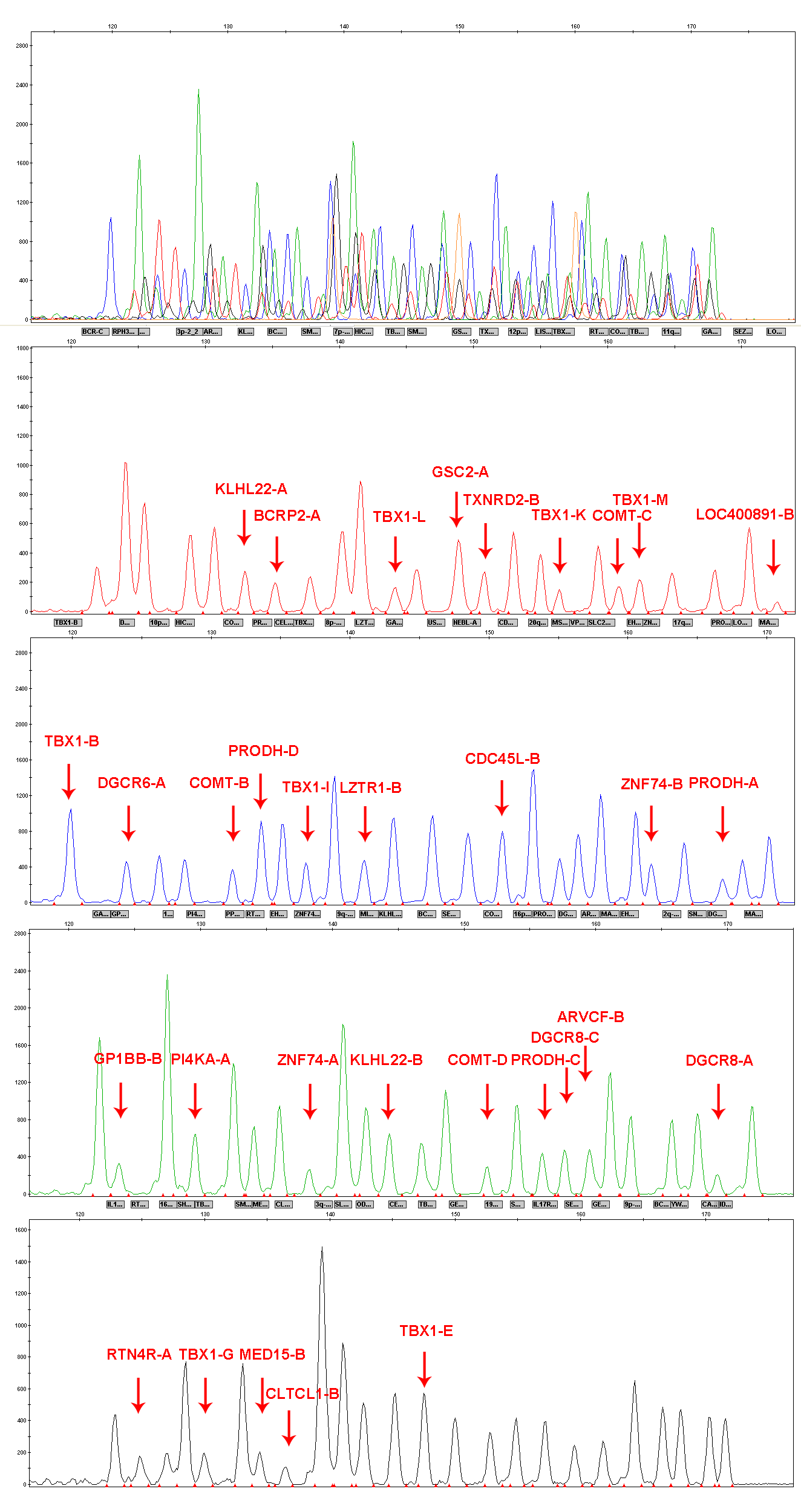
**

**22q11.2 deletion-Group 2**

**
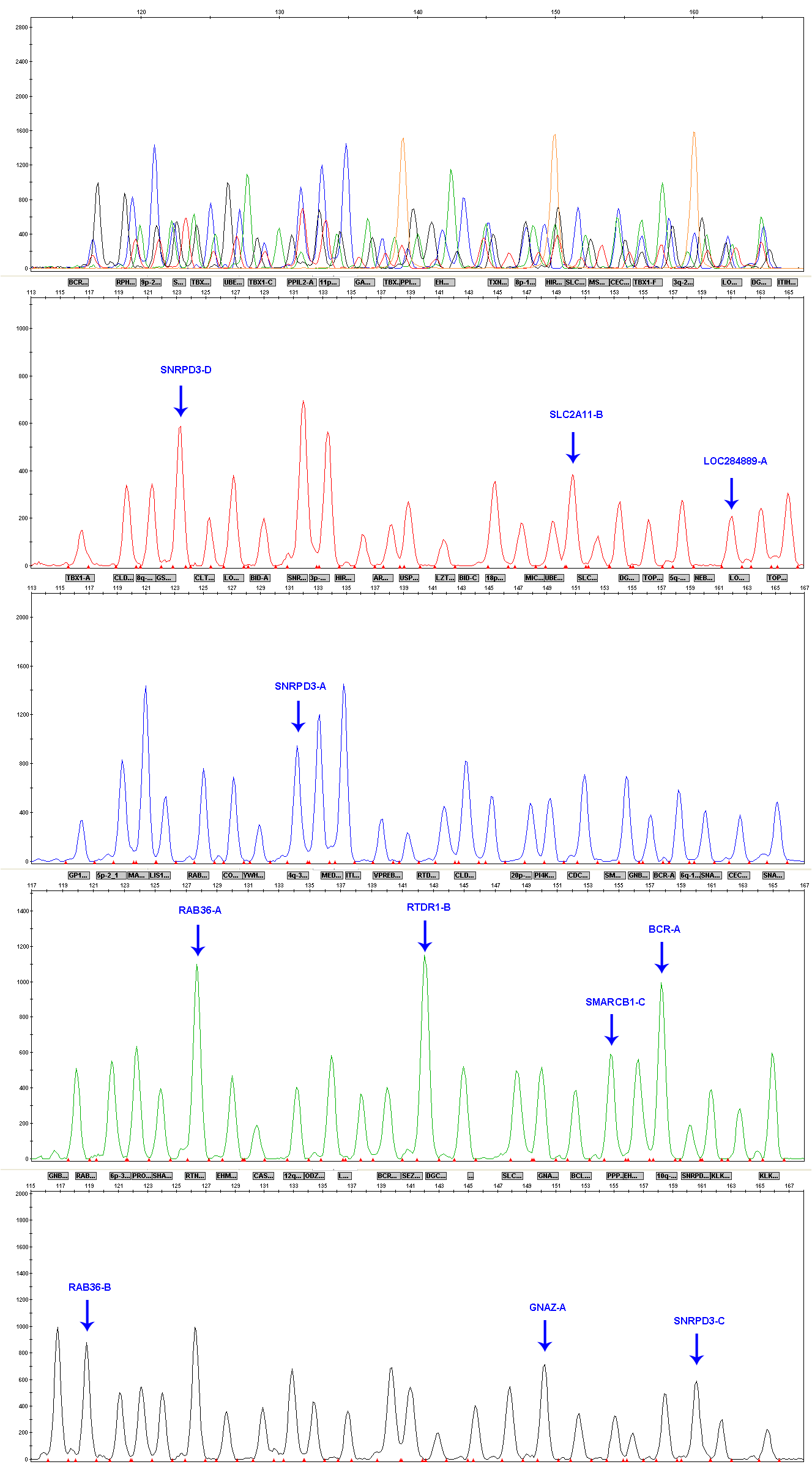
**

**22q11.2 duplication-Group 1**

**
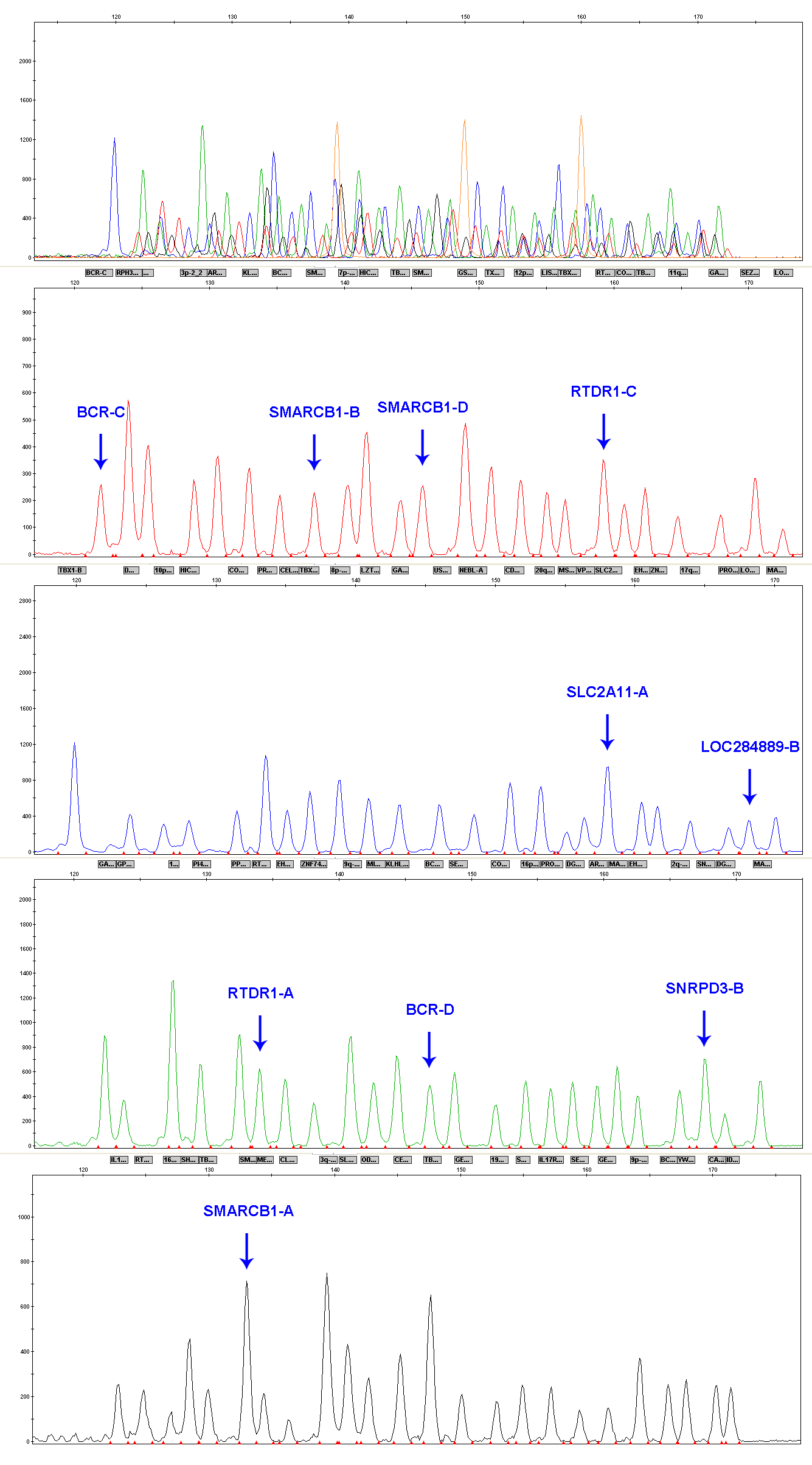
**

**22q11.2 duplication-Group 2**
